# Chronic kidney disease as a risk factor for peripheral nerve impairment in older adults: A longitudinal analysis of Health, Aging and Body Composition (Health ABC) study

**DOI:** 10.1371/journal.pone.0242406

**Published:** 2020-12-15

**Authors:** Simit Doshi, Ranjani N. Moorthi, Linda F. Fried, Mark J. Sarnak, Suzanne Satterfield, Michael Shlipak, Brittney S. Lange-Maia, Anne B. Newman, Elsa S. Strotmeyer

**Affiliations:** 1 Division of Nephrology, Indiana University, Indianapolis, IN, United States of America; 2 University of Pittsburgh, Pittsburgh, PA, United States of America; 3 Tufts Medical Center, Boston, MA, United States of America; 4 University of Tennessee, Memphis, TN, United States of America; 5 University of California, San Francisco, CA, United States of America; 6 Rush University Medical Center, Chicago, IL, United States of America; Szegedi Tudomanyegyetem, HUNGARY

## Abstract

**Introduction:**

Sensory and motor nerve deficits are prevalent in older adults and are associated with loss of functional independence. We hypothesize that chronic kidney disease predisposes to worsening sensorimotor nerve function over time.

**Materials and methods:**

Participants were from the Health, Aging and Body Composition Study (N = 1121) with longitudinal data between 2000–01 (initial visit) and 2007–08 (follow-up visit). Only participants with non-impaired nerve function at the initial visit were included. The predictor was presence of CKD (estimated GFR ≤ 60 ml/min/1.73m^2^) from the 1999–2000 visit. Peripheral nerve function outcomes at 7-year follow-up were 1) Motor: “new” impairments in motor parameters (nerve conduction velocity NCV < 40 m/s or peroneal compound motor action potential < 1 mv) at follow-up, and 2) Sensory: “new” impairment defined as insensitivity to standard 10-g monofilament or light 1.4-g monofilament at the great toe and “worsening” as a change from light to standard touch insensitivity over time. The association between CKD and “new” or “worsening” peripheral nerve impairment was studied using logistic regression.

**Results:**

The study population was 45.9% male, 34.3% Black and median age 75 y. CKD participants (15.6%) were older, more hypertensive, higher in BMI and had 2.37 (95% CI 1.30–4.34) fold higher adjusted odds of developing new motor nerve impairments in NCV. CKD was associated with a 2.02 (95% CI 1.01–4.03) fold higher odds of worsening monofilament insensitivity. CKD was not associated with development of new monofilament insensitivity.

**Conclusions:**

Pre-existing CKD leads to new and worsening sensorimotor nerve impairments over a 7-year time period in community-dwelling older adults.

## Introduction

Chronic kidney disease (CKD) is associated with decreased functional independence [1] falls [2, 3] frailty [4] and poor quality of life [5], representing accelerated aging [6]. Sensory and proprioceptive inputs to the central nervous system and appropriate motor outputs to the skeletal muscle are imperative to maintain balance and walking ability [7–11]. Therefore preserving peripheral sensory and motor nerve function may be important for maintaining normal posture and gait, preventing falls and fractures and to maintain the ability to live independently. Abnormal gait, falls, and fractures are common in patients with CKD [12–14] and in clinical practice it is noted that neuropathy increases the presence of all these outcomes [15]. In advanced CKD, as well as in patients who are dialysis dependent or end stage kidney disease, uremic neuropathy has been determined to be a progressive sensorimotor axonal neuropathy [15, 16]. In a prior cross-sectional analysis we have shown that early CKD in community dwelling older adults is associated with poor sensory nerve function [17]. However the role of early CKD in community dwelling older adults as a risk factor for worsening peripheral nerve function is unknown.

We hypothesized that CKD in older Black and white community-dwelling older adults is associated with the future development of new sensorimotor nerve function problems in participants without existing nerve deficits or worsening nerve function in those with existing deficits.

## Materials and methods

### A. Study population

**Fig 1:**
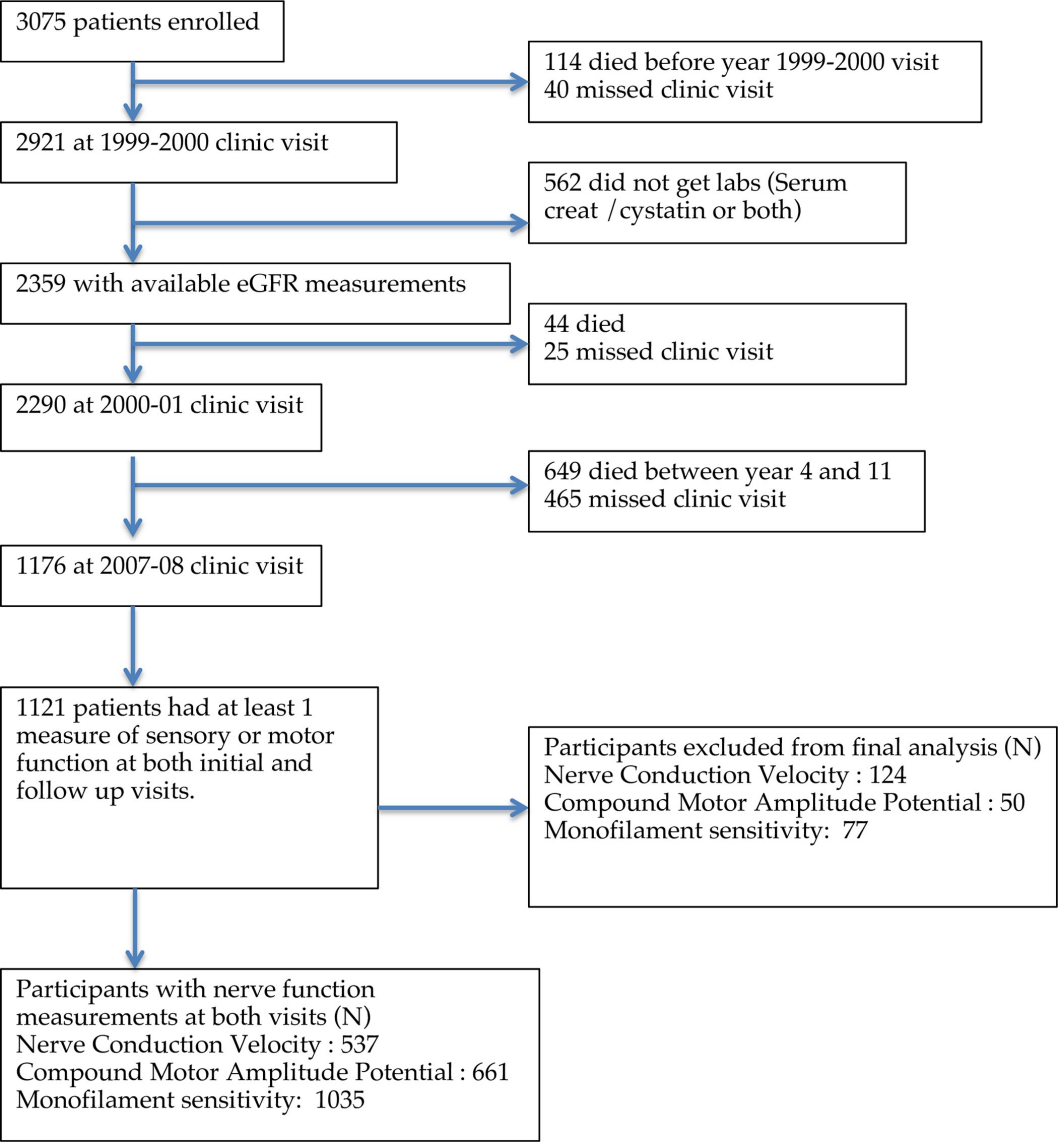
Participants included in analyses (N = 1121).

The Health Aging and Body Composition (ABC) study participants, (N = 3075, aged 70–79 years with no self-reported mobility disability at baseline), were recruited from April 1997 to June 1998 from two clinical locations in Memphis, TN and Pittsburgh, PA as previously described [18]. Eligible participants were able to walk a quarter mile and climb up 10 steps, planned to stay in the same geographic area for at least 3 years, and reported no life-threatening cancers treated in the 3 years before enrollment. At baseline, a detailed history, physical examination, laboratory measurements and body composition testing was performed. The study was approved by the Institutional Review Boards at the University of Tennessee Health Science Center and the University of Pittsburgh and all participants signed consent before participating. All participants included in this analysis had kidney function and at least 1 nerve measure available for both initial (2000–01) and follow up (2007–08) visits. (Fig 1) The current analysis was approved by the Institutional Review Board at Indiana University School of Medicine, Indianapolis IN.

### B. Measurements

#### Exposure variables

The glomerular filtration rate (eGFR) was estimated with the CKD-EPI creatinine-cystatin formula [19]. The estimated GFR at the 1999–2000 visit (was used to classify participants as having CKD (≤60 ml/min/1.73m^2^) or non-CKD (>60 ml/min/1.73m^2^). Serum creatinine was measured from blood collected after an overnight fast by a colorimetric assay using a Vitros 950 Analyzer (Johnson & Johnson, New Jersey, USA) calibrated to isotope-dilution mass spectrometry–traceable standards [20]. A particle-enhanced immunonephelometric assay on a BNII nephelometer (Siemens, Munich, Germany) was used to measure cystatin C [20].

#### Outcome variables

Motor and sensory peripheral nerve function in the lower extremity was evaluated at an initial 2000/2001 exam and 1 follow up exam in 2007/2008 by trained examiners at both clinical sites as described in prior study publications [11]. All testing was performed after warming extremities to ≥ 30°C and checking temperature at the beginning and end of the test.

*Motor function*. Motor amplitude assessed by compound motor action potential (CMAP) and motor nerve conduction velocity (NCV) was assessed at the peroneal nerve. Motor nerve conduction amplitude in millivolts (mV) was measured by stimulation at the popliteal fossa, fibular head and ankle using the NeuroMax 8 (XLTEK, Oakville, Ontario, Canada). Poor nerve function using clinical cut points for motor nerve amplitude and nerve conduction velocity have been shown to correlate with clinical neuropathy by Maser et al. [21]. We used cut off values of <1 mV and <40 m/s for CMAP and NCV respectively as has been done in prior publications of the HABC cohort [22]. Participants were considered to have transitioned from normal to poor if they met the predefined cut points [21, 22]. CMAP and NCV measures could be above or below the predefined cut offs at the initial visit, and thus the change categories [11] between initial (2000–01) and follow up (2007–08) visits were categorized as: (i) “Maintained Normal” (ii) “Normal transitioning to Poor” (iii) “Poor transitioning to Normal” and (iv) “Sustained Poor”, (S1 Table).

*Sensory function*. Sensory nerve function was measured as monofilament insensitivity, defined as the inability to detect at least 3 out of 4 touches at the dorsum of the large toe with a 1.4-g monofilament (light touch) or 10-g (standard touch, more severe). For monofilament testing, participants were considered transitioned if they felt stimulation at the initial exam but not at the subsequent exam [11]. The change in monofilament sensitivity between the initial exam and the follow up exam are described in Supplemental materials (S3 Table).

Self-reported symptoms of peripheral neuropathy in the lower extremities were collected during initial and follow up visits and included (1) numbness, "asleep feeling,” prickly feeling or tingling (2) sudden stabbing, burning, or deep aches on either foot or leg in the past 12 months.

Additional covariates included age, body mass index (BMI; (kg/m^2^), and self-reported sex, race, and smoking status (never, past or current) at year 2000–01. Diabetes was defined as a fasting plasma glucose level ≥126 mg/dL, 2-hour oral glucose tolerance test result >200 mg/dL, use of hypoglycemic agents or a self-reported history. Blood pressure was measured in the right upper extremity by certified staff using a conventional mercury sphygmomanometer. Hypertension was defined as systolic blood pressure >140 mmHg or diastolic blood pressure > 90 mmHg, taking anti-hypertensive medications, and/or a previous diagnosis by a physician of hypertension. Total calories spent walking and climbing stairs, as calculated from the administered modified Minnesota Leisure time Physical Activity Questionnaire, was used as a measure of physical activity [23]. A history of congestive heart failure or coronary artery disease (CAD) was classified as cardiovascular disease (CVD). Cerebrovascular disease was defined as a history of stroke or transient ischemic attack. Arm-ankle pressure index <0.9 was used to define peripheral arterial disease (PAD). Vitamin B 12 levels <260 pg/ml (Bayer HealthCare, Berkeley, California, USA) were considered low.

### C. Statistical analyses

Baseline characteristics of participants with versus without CKD were compared with 1) MannWhitney U-tests for continuous measures since these were not normally distributed and 2) chi- square tests for categorical measures. A two-sided p-value of <0.05 was considered significant. Chi-square tests compared the prevalence of nerve function parameters at the initial and the follow-up time points between those with CKD versus those without CKD. The proportion of those who transitioned categories of motor and sensory nerve function between CKD groups were also compared using Chi-square tests.

For motor variables of CMAP and the NCV, binomial logistic regression was used to determine the association between the presence of CKD and normal transitioning to poor motor nerve function. For this analysis, the reference group was “maintained normal” as defined above. For CMAP and NCV, the category of poor transitioning to normal had 1 and 5 participants respectively in the CKD group (S2A and S2B Tables) and was excluded from analyses. Furthermore, participants with poor function at initial visit could not develop a new impairment at follow-up visit and were excluded (N = 49 and 119 for CMAP and NCV resp.)

For sensory variable of monofilament insensitivity, multinomial logistic regression analysis was used. We conducted subgroup analysis for each group identified by categories of monofilament sensitivity at the initial visit (no deficit, light touch insensitivity, or standard touch insensitivity) as we could not assume equal prevalence and magnitude of risk of the covariates across initial categories of monofilament sensitivity.

For both motor and sensory analyses, covariates were chosen based on clinical relevance. For the adjusted logistic regression models we used a forward stepwise method (alpha level 0.05). Age, gender and race were forced into the fully adjusted model. Covariates were consistently applied to each of the outcome variables of CMAP, NCV and monofilament testing. Model fit was assessed by a likelihood ratio test and calibration was assessed using a cross-validated Hosmer-Lemeshow chi-square statistic. Collinearity of continuous predictors was tested in a linear regression model using variance inflation factor (VIF). A VIF >3 was considered a priori as significant collinearity but this condition was not met for any combination of variables.

SPSS 24.0 was used for all statistical analyses.

## Results

The cohort for the present analyses included 1,121 of the original 3,075 Health ABC Study participants who had had an initial kidney function measure at year 1999–2000 and nerve function measures at both initial (2000–01) and follow up (2007–08) visits (Fig 1). The median (IQR) age of analytic sample was 75 (73–78) years, 54.1% were women, median (IQR) eGFR was 75 (65–86) ml/min/1.73m^2^ and 15.2% had diabetes. Those with CKD (N = 175) compared to those without CKD (N = 946) were older, had higher BMI and were more likely to have hypertension (all p < 0.05; Table 1), but the prevalence of diabetes was similar. The proportion of women in the CKD (57.1%) versus non-CKD (53.5%) groups was not significantly different. Comparison of the analysis population (N = 1121) versus those not included in the final analysis (due to lack of nerve function measurements at two time points, N = 1954) is shown in S1 Table. The analyzed cohort was older, more likely to be white, and less likely to have diabetes, hypertension, and cardiovascular disease. Nerve function measurements and monofilament testing results at initial visit are as shown in Table 1.

**Table 1:** Demographic characteristics of participants (N = 1121) based on initial presence of CKD.

	Non-CKD	CKD	p value
	(>60 ml/min/1.73m^2^)	(≤60 ml/min/1.73m^2^)	
	N = 946	N = 175	
Demographics			
Age (Median, 25^th^ -75^th^ percentile)	74 (73–77)	76(73–78)	0.002
Male N (%)	440 (46.5)	75 (42.9)	0.37
Black N (%)	336 (35.5)	49 (28.0)	0.05
Lifestyle related			
Smoking at year 3 N (%)	49 (5.2)	6 (3.5)	0.42
Alcohol consumption (>1 drink/ week) N(%)	516 (54.7)	82 (46.9)	0.14
Body Mass Index (Median, 25^th^ -75^th^ percentile)	26.9 (24.1–29.7)	27.6 (25.1–30.4)	0.02
Comorbidities			
Diabetes N (%)	100 (10.6)	20 (11.4)	0.74
Hypertension N (%)	358 (38.0)	98 (56.0)	<0.001
Cardiovascular disease N (%)	182 (19.6)	46 (26.9)	0.06
Cerebrovascular disease N (%)	51 (5.4)	14 (8.1)	0.36
Peripheral Vascular Disease N(%)	98 (10.8)	25 (14.9)	0.29
Nerve measurements			
Initial CMAP, median (IQR)	3.4 (2.0–4.7)	3.2 (1.7–4.4)	0.17
Follow up CMAP, median (IQR)	1.3 (2.3–3.7)	1.9 (1.1–3.3)	0.04
Initial NCV, median (IQR)	44.0 (40.7–47.3)	43.4 (39.9–46.5)	0.09
Follow up NCV, median (IQR)	42.3 (39.1–45.1)	41.0 (37.9–43.7)	0.02
Monofilament testing			
Light touch insensitivity, initial	322 (34.3)	79 (45.1)	0.02
Standard touch insensitivity, initial	64 (6.8)	13 (7.4)	
Light touch insensitivity, follow up	290 (30.7)	51 (29.1)	0.02
Standard touch insensitivity, follow up	155 (16.4)	44 (25.1)	

• CMAP = Compound Motor Action Potential, NCV = Nerve Conduction Velocity, CKD = chronic kidney disease, Initial visit = year 2000–01, Follow up visit = year 2007–08

**Fig 2:**
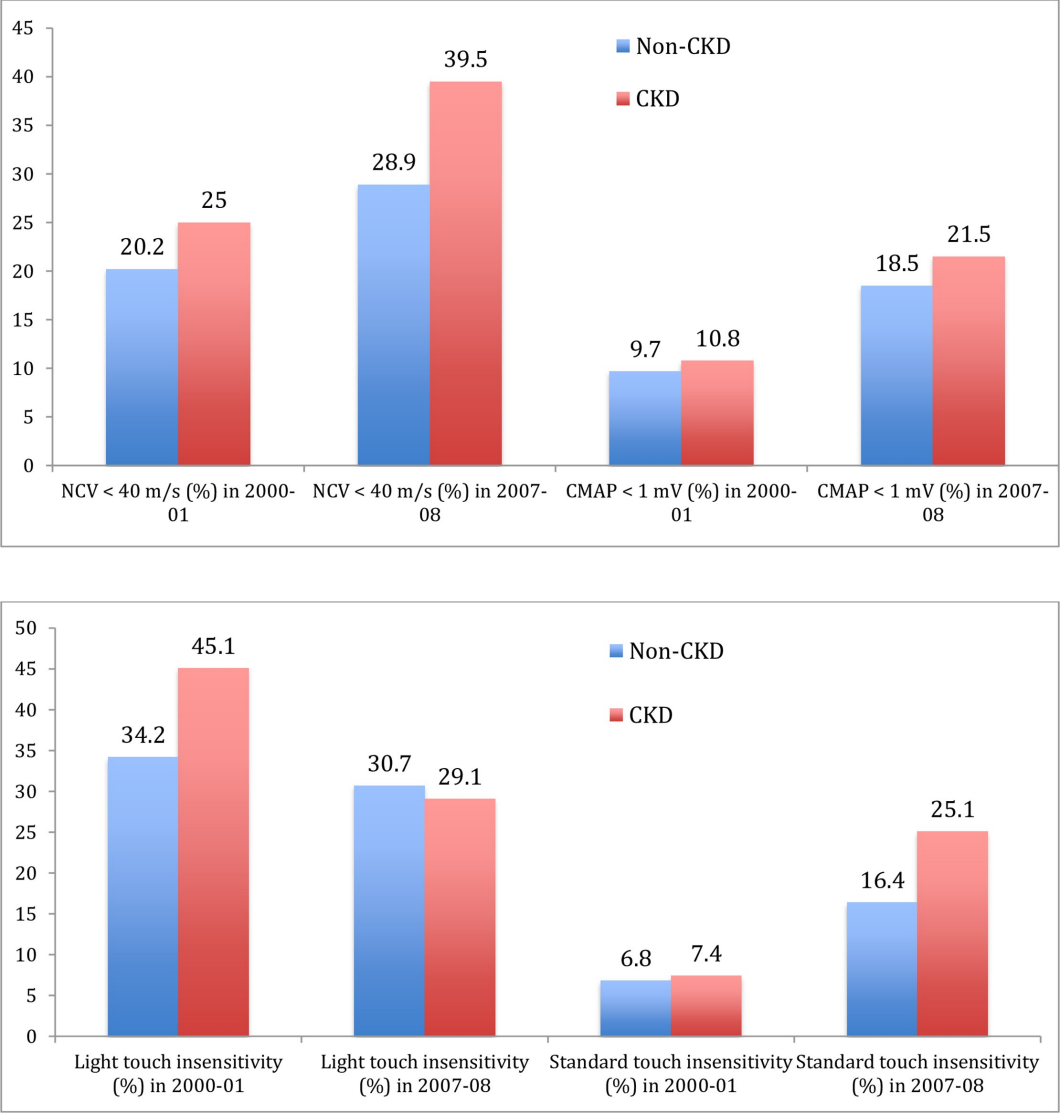
Point prevalence of motor (A) and sensory (B) Nerve function impairments at years 2000–01 and 2007–08 by presence of CKD in 1999–2000.

Among participants with available nerve measurements, CKD was noted in 93 (17.3%) participants with NCV measures available, 99 (15%) with CMAP measure and 175 (16.9%) with monofilament testing. (S2–S4 Tables) No significant differences were found in the mean initial motor CMAP or NCV between participants with and without CKD. Similarly, no significant differences existed in prevalence of initially poor CMAPs and NCV between those with and without CKD. At the follow up, visit no difference in prevalence of CMAP impairments existed between the groups but a higher prevalence of poor NCV were noted in the CKD population (39.5% vs 28.9%, p<0.05; Fig 2A). CKD participants were more likely to have transitioned to poor NCV over time (34.5% with CKD versus 20.1% without CKD, p = 0.005). No significant differences were found in participants with and without CKD for transition to poor CMAP. In the analysis cohort, CKD was noted in 26 (21.8%) of participants with new NCV deficits, 11 (13.3%) of new CMAP deficits, 22 (24.7%) of worsening monofilament testing and 39 (18.8%) of new monofilament testing.

Binomial logistic regression analyses (Table 2) showed that participants with CKD had 2.10 (95% CI 1.24–3.56) fold higher unadjusted odds of worsening NCV at follow up compared to the non-CKD group. This relationship remained significant (OR = 2.30; 95% CI 1.27–4.18) in an adjusted forward stepwise model with age, gender and race forced into the analysis.

**Table 2:** Relationship between pre-existing CKD and new deficit in motor function at follow-up.

NERVE FUNCTION	Unadjusted model	Adjusted model*
	OR and 95% CI	OR and 95% CI
New Amplitude deficit (CMAP<1 mV) N = 661	0.94(0.48–1.84)	0.85 (0.42–1.72)
New Velocity deficit (NCV <40 m/s) N = 537	2.10 (1.24–3.56)	2.30 (1.27–4.18)

*Adjusted model: Forward stepwise logistic regression with variables of NCV: Age, Race, Gender, BMI, cerebrovascular disease, cardiovascular disease, DM

CMAP: Age, Race, Gender, DM

CMAP = Compound Motor Action Potential, NCV = Nerve Conduction Velocity, CKD = chronic kidney disease, Initial visit = year 2000–01, Follow up visit = year 2007–08.

We did 3 separate sensitivity analyses for the outcome of new CMAP and NCV deficits. Logistic regression analysis was separately done in 520 participants who had no preexisting impairment in either NCV or CMAP and had both measures available at initial and follow up visits. Relationship of CKD status to new NCV and CMAP deficits remained consistent with primary analysis. Sensitivity analyses using eGFR as a continuous variable showed that lower eGFR increased the odds for developing of poor NCV. (OR per 10 ml/min/1.73 m^2^ lower eGFR:1.25, 95% CI 1.08–1.44). Sensitivity analyses with multivariate linear regression showed that a 10 unit decrease in eGFR was associated with a 0.146 m/s reduction in NCV (p = 0.002) after adjustment for covariates. In a similar analysis, eGFR was not associated with CMAP as a continuous outcome variable (p = 0.19)

Participants with CKD were more likely to have monofilament insensitivity to light touch (45.1% vs 34.3%) and standard touch (7.4% vs 6.8%) compared to those without CKD at initial visit (both p<0.05; Fig 2B). Higher prevalence of standard touch insensitivity was noted in the CKD group at follow up visit (25.1% vs 16.4%, p = 0.019). Symptoms of pain were reported by 32 (18.3%) CKD vs 157 (16.6%) non CKD (p = 0.59) participants at initial visit and 32 (18.3%) CKD vs 174 (18.4%) non CKD (p = 0.98) participants at follow up visit. Symptoms of numbness at were reported by 52 (29.7%) CKD vs 256 (27.1%) non-CKD (p = 0.46) at initial visit and 61 (34.9%) CKD vs 345 (36.4%) non CKD (p = 0.68) participants at follow up visit.

In multinomial logistic regression analyses including only participants with normal monofilament sensitivity initially, no significant differences were found for developing new monofilament insensitivity between those with and without CKD (Table 3). However, in participants with light touch insensitivity at initial visit, CKD conferred 2.09 times (95% CI 1.03–4.29) higher odds of progressing to standard touch insensitivity at the follow up visit. (Table 3)

**Table 3:** Multinomial regression analysis using CKD as predictor variable with outcome of “new” or “worsening” monofilament insensitivity.

Monofilament sensitivity	Unadjusted analysis OR and 95% CI	Adjusted analysis OR and 95% CI
Between years 2000–01 and 2007–08	CKD vs non-CKD	
**Participants who “maintained normal function” as reference category for outcome of “new” monofilament insensitivity** **#**		
Maintained normal function (N = 388)	Reference category	
New light touch insensitivity (N = 183)	1.41 (0.85–2.35)	1.51 (0.89–2.54)
New standard touch insensitivity (N = 64)	1.62 (0.79–3.39)	1.47 (0.70–3.08)
**Participants who “maintained function” as reference category for outcome of “worsening sensitivity” and “improving sensitivity”** **$**		
Maintained function (light touch insensitivity) (N = 136)	Reference category	
Worsened to standard touch insensitivity (N = 89)	1.79 (0.92–3.51)	2.09 (1.03–4.29)
Improved to normal sensitivity (N = 175)	1.42 (0.78–2.56)	1.64 (0.86–3.13)

# Forward stepwise model. Only participants with normal sensitivity at initial visit included

$ Forward stepwise model. Final model adjusted for age, gender, race, smoking status at year 3 and peripheral arterial disease. Reverse confounding variable for outcome of “worsened to standard touch insensitivity”: smoking and peripheral arterial disease

## Discussion

In this longitudinal study of a cohort of Black and white community-dwelling older men and women without initial nerve impairments, those with CKD had 2.3 times higher odds of developing worsened motor NCV over 7 years compared to those without CKD. Our finding persisted even when adjusted for known factors related to peripheral neuropathy, including older age, diabetes and BMI. In contrast, CKD was not associated with changes in CMAP. In a prior cross-sectional analysis in Health ABC participants, we found a 40% higher odds of impaired sensory function (monofilament insensitivity) in those with CKD. In the current study, CKD was also associated with a 2-fold higher risk of worsening from light to standard touch insensitivity during a 7 year follow up. Importantly, among participants with intact sensation at the initial visit, there was no difference in the development of new sensory impairments between those with and without CKD. Hence, CKD conferred additional risk for development of a new motor deficit (poor NCV) over time as well as worsening of preexisting sensory deficit (light to standard touch insensitivity).

Subjective symptoms would fail to identify such association in absence of objective testing in CKD. Objective measures of nerve function did not correlate with subjective symptoms of burning, tingling or numbness in our study. Other studies have also shown this discordance between symptoms and signs of nerve impairment in CKD and non-CKD populations [24, 25]. In a study of pre-dialysis patients, with median creatinine of 2.7+/- 0.6 mg/dl, 88% of participants had electrophysiological features but only 30% had symptoms suggestive of neuropathy [26]. This suggests that in CKD either the objective measures may precede the clinical symptoms, the clinical measures lack sensitivity, or that the symptoms are not only due to neuropathy.

Poor sensorimotor nerve function is commonly reported in patients with advanced CKD requiring dialysis [15]. In the present study we extend these findings and showed that early stage CKD, irrespective of diabetes, leads to progressive worsening of motor and sensory nerve function over time. We found that CKD was associated with worsening NCV but not significant worsening in CMAP. Mechanistically, NCV is maintained by myelin integrity while the motor amplitudes correspond to continuity of the axon and number of functioning axons [27]. The results in the present study, slowing of NCV without concurrent changes in CMAP, are a marker of demyelinating disease in early CKD. In other studies of patients with more advanced CKD and those on dialysis, large nerve fiber axonal degeneration with demyelination combined with axonal excitability is observed [15, 28] suggesting higher prevalence of abnormal markers of nerve function with more advanced CKD. Larger studies in diverse populations need to evaluate the natural progression of CMAP and NCV changes across different stages of CKD. In a cross sectional study of 100 patients with advanced CKD (mean eGFR 19.3+/- 8 ml/min) the NCVs correlated directly with decrease in renal function (p<0.01) [16]. Our results of worsening NCV over time in early CKD are consistent with NCV impairments observed in advanced CKD and dialysis patients in other studies [29, 30].

CKD has been associated with poor mobility evaluated as difficulty walking a predefined distance or climbing stairs and functional outcomes, similar to those described in older adults [14, 31, 32]. In our Health ABC participants, poor peripheral sensorimotor nerve function has been associated with increased time to complete 400-m walk test and worsening endurance over time [9]. Longitudinal assessment over 8.5 years of 2148 Health ABC participants with no mobility-disability in 2000/01 showed that poor peripheral sensorimotor function independently predicted self-reported mobility-disability (difficulty or inability to walk 0.25 miles or climb 10 steps) with incidence rate as high as 30% [11]. Poor sensorimotor function was also shown to be associated with lower quadriceps and ankle strength in 2059 participants with independent contribution from monofilament insensitivity [18]. In a systemic review of 6 cohorts in the aging population, peripheral nerve function was associated with poor mobility, even with adjustment for diabetes, although the nerve parameter assessed and the mobility measure varied in each study [33]. Significant correlation has been noted between indicators of cardiac autonomic function and motor nerve function parameters [34]. In Health ABC participants, poor NCV was associated with greater odds (OR = 1.6, 95% CI: 1.0–2.5) of postural hypotension whereas poor amplitude was associated with a higher resting HR. Poor NCV and CMAP have also been associated with lower bone mineral density (BMD) suggesting that patients with reduced peripheral nerve function may be more prone to fractures [35]. The long-term consequences of the development of poor nerve function on physical performance and cardiovascular health in those with early CKD should therefore be further evaluated.

Poor nerve function in patients with CKD is thought to be multifactorial in etiology. Elevated advanced glycation end product (AGE) formation has been associated with clinical neuropathy in both diabetes and non-diabetes populations with CKD [36, 37]. Other factors that potentially play a role in development of nerve alterations in CKD include hyperkalemia [36], hyperparathyroidism [29], and erythropoietin deficiency [38]. Interventions that have been shown to improve nerve function are limited, although a recent trial in patients with eGFR of 35+/- 8 ml/min demonstrated dietary potassium restriction improved the total neuropathy score over a 24-month period [39]. Erythropoietin [40] improved motor nerve conduction velocities in pre-dialysis patients with mean serum creatinine levels of 4.0 +/- 1.6 mg/dL. Establishing that a change in sensorimotor nerve function occurs with pre-existing CKD and identifying correlates, as we have in our analysis, is important in the design of future larger interventional studies for peripheral nerve impairments in earlier (pre-dialysis) CKD.

Strengths of our analysis were the size of the cohort and the longitudinal nature of the collection. Our study used objective, reliable and reproducible measures of motor nerve conduction as well as standardized clinical and subclinical sensory nerve assessments [22]. Limitations of the present study include the measure of kidney function performed one year prior to the initial peripheral nerve measures and that the definition of CKD did not discriminate between stable versus progressive CKD. Absence of urine studies as well as lack of repeat eGFR measurement at 3 months may have led to some misclassification of participants into the CKD or non-CKD categories. Assessment of sensory function was limited by absence of testing for warm and cold perception. The occurrence of new deficits especially in motor NCV testing was relatively low with only 12 new events identified in participants with CKD. Repeat measures for motor testing were not available in the entire analysis population. Finally, only two nerve function assessments were done during the follow up period. More assessments during the follow up period may also have allowed detection of more subtle changes and trends in nerve function.

In summary, we found that CKD is associated with worsening sensorimotor nerve function over a 7-year period in a cohort of community dwelling older adults. In the aging population without known CKD, such nerve problems contribute to incident mobility disability, falls, poor bone mineral density, physical activity, impaired activities of daily living and limiting functional independence [9, 11, 18, 25, 35, 41]. Thus, it is plausible that the poor nerve function in early stage CKD observed in the present study may be an important risk factor for the widespread morbidity and impaired physical mobility that is observed in this population. Future studies in patients with CKD should confirm this association and determine if early detection of peripheral nerve impairments may lead to preventive interventions to reduce falls and mobility decline.

## Supporting information

S1 Table Analysis population compared to participants excluded from analysis from the entire Health ABC population (N = 3075). (DOCX)

S2 Table NCV changes over time (N = 537). (DOCX)

S3 Table CMAP changes over time (N = 661). (DOCX)

S4 Table Monofilament testing over time (N = 1035). (DOCX)

S5 Table Symptoms at initial visit and follow up years for all participants (N = 1121). (DOCX)
