# Supplementary material for: Chronic kidney disease as a risk factor for peripheral nerve impairment in older adults: A longitudinal analysis of Health, Aging and Body Composition (Health ABC) study
Source: PLoS One. 2020 Dec 15;15(12):e0242406. doi: 10.1371/journal.pone.0242406 (PMC7737903; doi:10.1371/journal.pone.0242406)
Supplement: S1 Table — (DOCX) [file pone.0242406.s001.docx]

S1 Table. Analysis Population compared to participants excluded from analysis from the entire Health ABC population ( N=3075)

|  | Analysis population  (N=1121) | Excluded  Participants  (N=1954) | P value |
| --- | --- | --- | --- |
| Demographics |  |  |  |
| Age Mean(SD) | 75(3) | 73 (3) | <0.001 |
| Male (%) | 515 (45.9) | 976 (49.9) | 0.04 |
| Black (%) | 385 (34.3) | 897 (45.9) | <0.001 |
| Lifestyle related |  |  |  |
| Smoking history (%) | 55 (5.0) | 265 (13.6) | <0.001 |
| Alcohol consumption (>1 drink/ wk) (%) | 598(53.4) | 927 (47.4) | 0.002 |
| BMI Mean (SD) | 27.4 (4.6) | 27.4 (4.8) | 1.0 |
| Comorbidities |  |  |  |
| Diabetic (self-report or meds) (%) | 120 (10.7) | 347 (17.8) | <0.001 |
| Hypertension (self-report or meds) (%) | 456 (40.8) | 909 (46.5) | <0.001 |
| Cardiovascular disease (%) | 228 (20.7) | 532 (27.2) | <0.001 |
| Cerebrovascular disease (%) | 65 (5.8) | 157 (8.0) | 0.03 |
| Peripheral Vascular Disease (%) | 45 (4.1) | 118 (6.0) | 0.02 |
