# Supplementary material for: Chronic kidney disease as a risk factor for peripheral nerve impairment in older adults: A longitudinal analysis of Health, Aging and Body Composition (Health ABC) study
Source: PLoS One. 2020 Dec 15;15(12):e0242406. doi: 10.1371/journal.pone.0242406 (PMC7737903; doi:10.1371/journal.pone.0242406)
Supplement: S2 Table — (DOCX) [file pone.0242406.s002.docx]

S2 Table NCV changes over time (N=537)

| Non-CKD N (%), N=444) | | | |
| --- | --- | --- | --- |
| Year | 2007-08 (follow up) | | |
| 2000-01 (initial visit) |  | Normal | NCV <40 m/s |
|  | Normal | 369 (65) | 93 (16) |
|  | NCV < 40 m/s * | 34 (6) | 72 (13) |

| CKD N (%) , N=93 | | | |
| --- | --- | --- | --- |
|  | 2007-08 (follow up) | | |
| 2000-01 (initial visit) |  | Normal | NCV <40 m/s |
|  | Normal | 49 (53) | 26 (28) |
|  | NCV < 40 m/s * | 5 (5) | 13 (14) |

* = excluded from analysis due to preexisting impairments
