# Supplementary material for: Chronic kidney disease as a risk factor for peripheral nerve impairment in older adults: A longitudinal analysis of Health, Aging and Body Composition (Health ABC) study
Source: PLoS One. 2020 Dec 15;15(12):e0242406. doi: 10.1371/journal.pone.0242406 (PMC7737903; doi:10.1371/journal.pone.0242406)
Supplement: S3 Table — (DOCX) [file pone.0242406.s003.docx]

S3 Table CMAP changes over time (N=661)

| Non-CKD N (%), N=562 | | | |
| --- | --- | --- | --- |
| Year | 2007-08 (follow up) | | |
| 2000-01 (initial visit) |  | Normal | CMAP < 1 mV |
|  | Normal | 497 (81) | 72 (12) |
|  | CMAP < 1 mV * | 12 (2) | 31 (5) |

| CKD N (%), N=99 | | | |
| --- | --- | --- | --- |
|  | 2007-08 (follow up) | | |
| 2000-01 (initial visit) |  | Normal | CMAP < 1 mV |
|  | Normal | 81 (81) | 11 (11) |
|  | CMAP < 1 mV * | 1 (1) | 6 (6) |

* = excluded from analysis due to preexisting impairments
