# Supplementary material for: Chronic kidney disease as a risk factor for peripheral nerve impairment in older adults: A longitudinal analysis of Health, Aging and Body Composition (Health ABC) study
Source: PLoS One. 2020 Dec 15;15(12):e0242406. doi: 10.1371/journal.pone.0242406 (PMC7737903; doi:10.1371/journal.pone.0242406)
Supplement: S4 Table — (DOCX) [file pone.0242406.s004.docx]

S4 Table: Monofilament testing over time (N=1035)

| Non-CKD N(%) | | | | |
| --- | --- | --- | --- | --- |
| Year | 2007-08 (follow up) | | | |
| 2000-01 (initial visit) |  | Normal | Light touch insensitivity | Standard touch insensitivity |
|  | Normal | 344 (37) | 155 (17) | 53 (6) |
|  | Light touch insensitivity | 139 (14) | 115 (12) | 67 (7) |
|  | Standard touch insensitivity | 10 (1) | 19 (2) | 35 (4) |

| CKD N (%) | | | | |
| --- | --- | --- | --- | --- |
| Year | 2007-08 (follow up) | | | |
| 2000-01 (initial visit) |  | Normal | Light touch insensitivity | Standard touch insensitivity |
|  | Normal | 44 (25) | 28 (16) | 11 (6) |
|  | Light touch insensitivity | 36 (21) | 21 (12) | 22 (12) |
|  | Standard touch insensitivity | 0 (0) | 2 (<1) | 11 (6) |
