# Supplementary material for: Chronic kidney disease as a risk factor for peripheral nerve impairment in older adults: A longitudinal analysis of Health, Aging and Body Composition (Health ABC) study
Source: PLoS One. 2020 Dec 15;15(12):e0242406. doi: 10.1371/journal.pone.0242406 (PMC7737903; doi:10.1371/journal.pone.0242406)
Supplement: S5 Table — (DOCX) [file pone.0242406.s005.docx]

S5 Table. Symptoms at initial visit and follow up years for all participants (N = 1121)

|  | Non-CKD  (>60 ml/min/1.73m^2^)  N=946 | CKD  (≤60 ml/min/1.73m^2^)  N=175 | p value |
| --- | --- | --- | --- |
| Symptoms (year 2000-01 (initial visit)) |  |  |  |
| Stabbing pain N(%) | 157 (16.6) | 32 (18.3) | 0.59 |
| Numbness N(%) | 256 (27.1) | 52 (29.7) | 0.46 |
| Symptoms (year 2007-08 (follow up)) |  |  |  |
| Stabbing pain N(%) | 174 (18.4) | 32 (18.3) | 0.98 |
| Numbness N(%) | 345 (36.4) | 61 (34.9) | 0.68 |
